# Evaluation the dentists’ awareness of inherited bleeding disorders and anticoagulants in Shiraz

**Published:** 2013-10-22

**Authors:** R Robati, MM Farokhi

**Affiliations:** **1. Department of Microbiology, Science and Research branch, Islamic Azad University, Fars, Iran**

**Keywords:** Knowledge, Blood Coagulation Tests, Blood Coagulation Disorders

## Abstract

**Background:**

Some of the dental procedures can cause bleeding. Bleeding control can be difficult in some patients because of systemic disease or chronic anticoagulant therapy, so they may be at increased risk for bleeding occurrences or even death following invasive dental procedures. This study was schemed to measure the knowledge of general dentists in Shiraz city regarding coagulation tests performed in bleeding disorders during 2011 to 2012.

**Materials and Methods:**

In this descriptive-analytical study, a questionnaire (consist 23 questions) was designed with the help of specialists in the field of oral medicine and hematology. This questionnaire was distributed among 146 general practitioners. Collected information was analyzed with SPSS version 16.

**Results:**

The mean mark for dentist’s knowledge was 9.40 ± 1.30 (categorized moderate level). There was no significant difference in the mean knowledge scores among male and female dentists. Tukey’s test displayed a significant difference in the mean knowledge level among 21 to 30 years old and over forty years' old dentists (p< 0.03).

**Conclusions:**

This study showed that knowledge of the dentists regarding bleeding disorders is not at optimal level which needs planning for continuing education courses.

## Introduction

Bleeding disorders include conditions in which ability of blood vessels, platelets, and coagulation agents to establish hemostasis is changed (1). These disorders are divided into acquired and inherited categories. Acquired coagulation disorders are caused by intake of certain medicines and special systemic diseases [2]. Von Willebrand disease is the most common inherited coagulation disorder which results from deficiency of Von Willebrand factor and involves nearly 1% of society’s population (2). Once the patients suffering from coagulation disorders undergo some dental treatments which cause bleeding, the questions concern the tests to be requested for the patient prior to dentistry interventions and the changes that shall be applied to anticoagulants, and also, the allowed dental treatments for these patients. The reason is the fact that physicians under such circumstances must assess the ability of patients to reach post-operative hemostasis as well as likelihood of thrombosis and emboli occurrence (3). Through a study on 4163 individuals aging over 65 years in North Carolina, 51.7% of the respondents used to receive anticoagulants and were potentially susceptible to hemostatic system suppression (2).

In a research carried out by Salehi (2006) in Esfahan City on level of general dentists’ awareness about coagulation tests in patients suffering from coagulation diseases, it was concluded that dentists’ knowledge about dentistry procedures in patients suffering from bleeding disorders and requiring pretreatment coagulation tests was at a suitable level (4).

Through a research by Linebour et al. (2007) entitled “Instructional Methods concerning Anticoagulants and Dentistry Operations in USA Dentistry Faculties”, 28 dentistry faculties (50.9%) responded to the questions. Unlike the available evidences that suggested no need to modify the anticoagulants, most of dentistry faculties responded that they teach dentistry students to talk with the patients about modification of treatment with Warfarin for many of routine dental operations (including: dental plaque removal: 21.4%, dental restoration: 14.3%, extraction of single-root tooth: 46.6%, extraction of multiple-root tooth: 64.3%, and root treatment: 17.9%). Nonetheless, 67.9% responded that international normalized ratio (INR) between 2 and 3 is acceptable for dentistry procedures. Ultimately, some contradictions are observed between academic activities in USA dentistry faculties and medical documents (5).

Taking into account relatively high virulence and also availability of limited similar studies concerning level of dentists’ awareness of coagulation tests, the present study analyzed level of general dentists’ knowledge about coagulation tests in bleeding disorders in order to evaluate effectiveness of instructional methods and also their updated information through analysis of the acquired results.

## Materials and Methods

Taking into account significance of hemorrhage subject in diverse dental operations and necessity of dentists’ familiarity with common bleeding disorders, anticoagulants, and blood coagulation screen tests, a questionnaire was codified in a descriptive-analytical study to assess the awareness level of dentists. This questionnaire was designed based on similar questionnaires in this area (Salehi’s study in Esfahan) and also via consultation with an oral medicine specialist as well as a hematologist (4). Subsequently, pilot study was conducted on 10 general dentists and reliability and validity of the questionnaire were confirmed. The questionnaire consisted of two sections (personal specifications and awareness) and 23 questions. Due to significance of hemorrhage issue in diverse dentistry procedures, questions of the questionnaire were designed based on common bleeding disorders, anticoagulants, and blood coagulation filtering tests. After referring to Medicine Deputy of Shiraz University of Medical Sciences, list of names of general dentists was obtained. The questionnaires were then distributed among the respective dentists. The questionnaires were filled through personal presence and referral to offices of the dentists. The dentists were excluded from research in the case of unwillingness to collaborate.

Out of 164 general dentists, 146 individuals filled the aforementioned questionnaire. The questionnaire data was prepared for statistical analyses. For this purpose, some of variables were categorized as below:

Age variable was divided into 3 categories: below 30 years, 31 to 40 years, and over 40 years. Variable of post-graduation duration was also classified into 4 categories: below 5 years, 5-10 years, 10-15 years, and more than 15 years. Last dentist’s study about coagulation tests was classified in 3 groups: less than 5 years, 5 to 10 years, 10 to 15 years, and over 15 years. 

Questionnaire responses were specified according to credible references in this field (1, 2, and 4). A positive point was considered for every correct answer and a zero score was assigned to every incorrect (false or blank). Finally, sum of awareness score for dentists was assumed to be 23. For ranked evaluation of awareness level, sum of dentists’ scores acquired from responding to the questions were used. Thus, awareness was categorized into three levels: poor (0 to 8), moderate (9-16), and good (17-23). 

**Statistical Analysis**

Questionnaire data was analyzed using statistical tests including: t-test, ANOVA, and Tukey’s multiple comparisons. 

## Results

In the present research, dentists’ awareness of dentistry procedures for individuals suffering from bleeding disorders was assessed to be at a moderate level (69.26%).

Response percentage of dentists to questionnaires was 89%. 91 individuals or 62.2% of participating dentists were male and the rest 55 persons (37.8%) were female. Average age of dentists was 34.3 ± 5.9 years (with variation range of 24 to 65 years) (Table I).57.3 % of dentists replied correctly to the question concerning Bleeding time (BT), which highlight need for further notification in this respect. 

Keeping in mind the rating of dentist’s awareness level in terms of the score acquired from responses to the questions, awareness level of 85 persons (58.2%) was assessed to be moderate (score: 9 to 16). 48 persons (32.8%) had poor awareness (score 0 to 8) and the rest 13 persons (8.9%) had good awareness (score 17 to 23) of coagulation disorders (Table I).

Average awareness score of female and male dentists was 9.21 ± 1.05 and 8.45 ± 1.04(TableI). Average awareness scores of two groups of dentists did not have statistical significant difference.

There was a significant statistical difference in terms of awareness score among three age groups of dentists (p<0.04). Tukey’s test showed that average awareness had significant statistical difference only between age groups of 31 to 40 years and over 40 years. 

There were no significant statistical contrasts between age groups of over 40 years and below 30 years or between age groups of below 30 years and 31-40 years.

One-way ANOVA test did not reveal a significant difference in analysis of mean values of dentists’ awareness scores with respect to the post-graduation duration.

**Figure 1:**
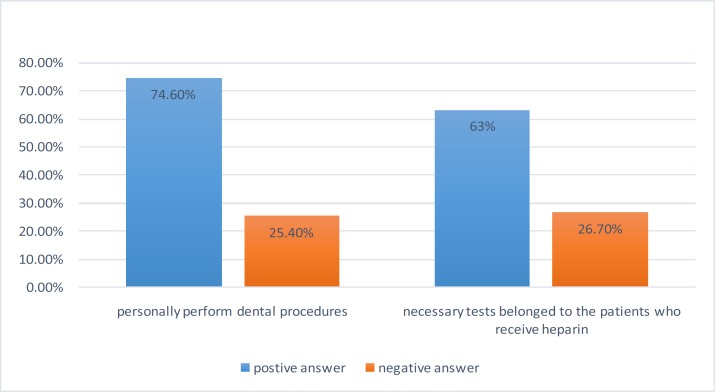
Comparing of positive and negative responses to personally perform dental procedures and necessary tests belonged to the patients who receive heparin

Majority of dentists (77.6%) stated a time less than 5 years as an answer to the question: “when was the last time you studied about coagulation tests?” and mentioned books as the main source of their information acquisition. In answer to the question: “would you personally perform dental procedures in the case of suspicion to bleeding disorders in the patients referring to your office?” 109 persons (74.6%) responded affirmatively and the rest 37 dentists (25.4%) stated that they would perform no dental procedure in these cases (Figure 1). The highest level of dentists’ awareness about the necessary tests belonged to the patients who receive heparin. 63.3% of dentists replied correctly to this question. Therefore, their awareness level was assessed to be good (chart 1). The lowest awareness level of general dentists (25.8%) was observed in maximal value of Partial thromboplastin time (PTT) test for dentistry procedures. Level of dentists’ awareness about anticoagulants was poor (14.3%). Their knowledge about dentistry procedures in patients with bleeding disorder was relatively good (76.2%).

Maximal Prothrombin time (PT) values in dental operations are up to 1.5 times of normal value (11 to 15 seconds with an average of 40 seconds), BT up to 2.5 times of normal value (15 minutes), and PTT equal to 25-35 seconds (an average of 75 seconds). But unfortunately, dentists’ awareness levels in this regard were respectively estimated 32.1%, 30.3%, and 29.6%, reflecting low awareness level.

**Table I:** percentage and frequency of gender, Responsibility to questionnaires and awareness level

Average ± Standard Deviation Frequency Percentage		
34.3 ± 5.9 Age (years)		
102 62.2 62 37.8	Male Female	Gender
130 89 16 11	Response Not response	Responsibility To questionnaires
48 32.8 85 58.2 13 8.9	Poor Average Good	Awareness Level

## Discussion

Some of dentistry operations can cause bleeding. Under normal circumstances, low risk threatens the patients but risk of bleeding events or even death is more pronounced in patients whose bleeding control is impaired due to suffering from certain systemic diseases or administration of some medicines unless the dentist realizes their problem before performing any operation. Most often, special measures are taken as soon as identifying patients suffering from bleeding disorder in order to mitigate hemorrhage risk during dental operations. The tests needed for assessment of bleeding disorders are demanded when the patients declare personal or familial record of bleeding in the past and/or symptoms of bleeding disorders are detected through the clinical examinations.

Higher level of awareness in nurses aging less than 40 years could result from more updated information of young dentists and also modifications in contents of reference books during recent years.

Most correct answers belonged to periodontal operations (100%) and least correct answer was reported for stitch removal (21%). Hence, owing to similarity of questions in the present questionnaire and Salehi’s research, it can be asserted that the results of the current research are in disagreement with Salehi’s findings such that the latter study assigned the most and least correct answers in general dentists of Esfahan city to implant and plaque and intramuscular injection (4).

Concerning minimal number of platelets for performing all dental operations, 56.8% of general dentists replied the respective question correctly, which is indicative of need to promotion of their awareness level in this respect. In this regard results of the present research are very close to Salehi’s findings (48.5%) (4).

According to our results, it seems vital to promote awareness level of dentists in this regard due to significance of dentistry measures in systemic patients including in bleeding disorders (6 , 7). In terms of the aforementioned variables, the results of the present research were consistent with Salehi’s findings because he also implied low awareness level of dentists of Esfahan City in this respect (4).

PT and PTT values are obtained via adding a thromboplastic agent to plasma. Discrepancy in thromboplastic activity might result from different PT values for a single plasma sample. Thus, accurate evaluation of anticoagulant activity level solely based on PT is difficult. Therefore, in order to lower the discrepancies, International Normalized Ratio (INR) is currently the selected method for reporting PT values.Only 34.2% of general dentists in the current research were aware of normal values of INR test. 

The reason might be the fact that responses to formerly used PT test differed in different regions because of conducting the respective tests with different kits and procedures. Consequently, more experienced dentists seem to have less information about such matters (7, 8). As a result, dentists must be informed about this new test through various instructional programs (4).

60.2% of dentists replied correctly to the question about coagulation tests in users of anticoagulants. Considering widespread application of these medicines, it seems that dentists must be sufficiently aware of conducting necessary tests for the respective patients. Nonetheless, Salehi’s study suggested lower awareness level (34.3%) of dentists in Esfahan.

Aspirin is a common medicine used on a daily basis for a long time by many people, especially those suffering from coronary artery obstructions and/or patients suffering from inflammatory diseases such as rheumatoid arthritis. Since aspirin affects performance of platelets. In the case of need to terminate medicine for reduction of BT values or prevent from potential severe bleeding after performing BT test, termination, reduction or substitution of medicine shall be indeed implemented after consulting with the doctor. Minimal duration for medicine termination is 3 days (8-12). In the present study, 53.7% of dentists mentioned 3 days as their answer to this question. Considering widespread application of these medicines, it seems that the dentists must be sufficiently aware of conducting the needed tests for the respective patients.

Warfarin and coumarin are commonly used oral anticoagulants prescribed for patients suffering from coronary disorders resulting from arterial obstruction due to clot formation. Where necessary, after conducting PT tests, these medicines must be terminated or regulated in accordance with physician’s advice at least for 3 days because they might increase bleeding during the dental operations that are accompanied with hemorrhage (10, 11, and 13). In the present study, 59.9% of general dentists had sufficient knowledge in this regard and chose the right answer.

People suffering from heart problems associated with coronary artery obstructions or dialysis patients take heparin for prevention from clot formation. Since half-life of heparin is approximately 3-4 hours, the dental operations must accordingly start at least 6 hours after heparin administration time. Yet occasionally, due to probable changes, the ideal time for implementing dental procedures is considered 24 hours after heparin administration in these patients (11). In the present study, 54.2% of dentists stated 24 hours as the initiation time of dental procedures, which might result from lack of attention to the minimal time elapsed for heparin half-life. Salehi estimated a similar awareness level for Esfahan’s dentists in this respect as well (4).

To reach local sedation in patients suffering from coagulation disorders, the coagulation factors need to be at the ideal level such that minimal value of coagulation factors for neural block sedation is 30%. 

51.3% of dentists replied correctly to this question. Concerning the patients suffering from coagulation disorders, some local sedative injections (such as intra-ligament injection) is admissible and there is no need for correction of coagulation factor. 50.2% of patients answered this question correctly. Results of the current research concerning dentists’ awareness of local sedation in the respective patients were in agreement with Salehi’s findings. 

Taking into account the fact that subject of bleeding disorders have been so far instructed by dentistry faculty members during PhD program of general dentistry, unawareness of dentists seems to result from limited number of credits and insufficient hours dedicated to the respective course. In the light of awareness and performance shortcomings of dentists, Academic Planning Council of Medical Sciences in 2011 ratified revision of curriculum or academic syllabus of general dentistry’s PhD program. It was thereby decided that the course “systemic diseases” (including blood and bleeding diseases) to be added to curriculum of dentistry with 2.5 credits. It is therefore hoped that awareness level of dentistry students or future dentists of Iranian society be promoted through implementation of the new curriculum.

## Conclusion

The present research indicated that dentists’ awareness of bleeding disorders is at a desirable level. But it is necessary to hold codified instructional programs and to provide the needed knowledge in this respect to students as well as to the dentists currently working in the cities.

## Conflict of Interest

The authors have no conflict of interest.
